# Supplementary material for: Microscale cavitation as a mechanism for nucleating earthquakes at the base of the seismogenic zone
Source: Nat Commun. 2017 Nov 21;8:1645. doi: 10.1038/s41467-017-01843-3 (PMC5696358; doi:10.1038/s41467-017-01843-3)
Supplement: Supplementary file 1 — Supplementary Information [file 41467_2017_1843_MOESM1_ESM.pdf]

Supplementary Table 1. List of experiments and conditions.

Experiment	*v (μm s^{-1})*	*σ_n^{eff} (MPa)*	*P_f [1] (MPa)*	*T (°C)[2]*	*x_{fnl} (mm)[3]*
550-slow	0.1	50	100	541±2.3	7.90
550-fast	100	50	100	552±0.6	10.4
NS-slow_A	0.1	50 | 65 | 80 | 100 | 120 | 140	100	548±3.7	12.6
NS-slow_B	0.1	50 | 65 | 80 | 100 | 120	100	549.1±3.5	29.7
NS-fast_A	10	30 | 50 | 80 | 100	100	544±0.6	12.4
NS-fast_B	10	50 | 65 | 80 | 100	100		
NS-fast_C	1	50 | 65 | 80 | 100 | 120	100	547.2±0.7	26.2
VS-slow	0.1 | 0.03 | 0.01 | 0.003 | 0.001	50	100	551.7±1.1	8.50
VS-fast	1 | 0.5 | 0.3 | 1 | 0.3 | 0.1 | 1 | 3 | 10 | 30 | 100	50	100	555.7±0.1	9.45

[1]Pore fluid pressure.
[2]Mean temperature measured over the duration of a test, with its standard deviation.
[3]Total shear displacement.

Supplementary Table 2. List of shear strength values constant *v* experiments.

Experiment	*v* (*μm s*$^{-1}$)	σ_n^{eff} (*MPa*)	P_f (*MPa*)	*T* (°C)	τ_{ss}[1)] (*MPa*)	μ_{ss}[2)]
550-slow	0.1	50	100	541±2.3	37.8	0.76
550-fast	100	50	100	552±0.6	23.4	0.47
NS-slow_A	0.1	50	100	548±3.7	41.3	0.83
		65			49.2	0.76
		80			53.1	0.66
		100			55.5	0.55
		120			59.3	0.49
		140			n/a[3)]	n/a[3)]
NS-slow_B	0.1	50	100	549.1±3.5	36.3	0.73
		65			45.4	0.70
		80			54.2	0.68
		100			60.7	0.61
		120			64.5	0.54
NS-fast_A	10	30	100	544±0.6	17.6	0.59
		50			30.2	0.60
		80			50.1	0.63
		100			60.6	0.76
NS-fast_B	10	50	100	539±0.5	26.6	0.53
		65			35.5	0.55
		80			41.9	0.52
		100			48.9	0.49
		120			36.4	0.40
NS-fast_C	1	50	100	547.2±0.7	30.4	0.61
		65			39.8	0.61
		80			49.5	0.62
		100			61.2	0.61
		120			72.8	0.61

[1)]Steady-state shear strength, except In the case of stick (in grey), for which we report the maximum stress before failure.
[2)]Apparent coefficient of friction at steady state, defined $\mu_{ss} = \tau_{ss}/\sigma_n^{eff}$.
[3)]Steady-state sliding was not reached in this interval due to furnace failure.

Supplementary Table 3. List of shear strength values *v*-stepping experiments.

Experiment	*v (μm s⁻¹)*	σ_n^{eff} *(MPa)*	P_f *(MPa)*	*T (°C)*	τ_{ss}[1] *(MPa)*	μ_{ss}[2]
VS-slow	0.1	50	100	555.7±0.1	36.6	0.73
	0.03				36.1	0.72
	0.01				31.9	0.64
	0.003				23.9	0.48
	0.001				15.8	0.32
VS-fast	1	50	100	551.7±1.1	28.5	0.57
	0.5				28.6	0.57
	0.3				28.8	0.58
	1				28.2	0.56
	0.3				29.1	0.58
	0.1				30.0	0.60
	1				28.8	0.58
	3				26.6	0.53
	10				24.2	0.48
	30				22.3	0.45
	100				20.0	0.40

[1](Near-)steady-state shear strength measured at the end of an interval.
[2]Apparent coefficient of friction at steady state, defined $\mu_{ss} = \tau_{ss}/\sigma_n^{eff}$.

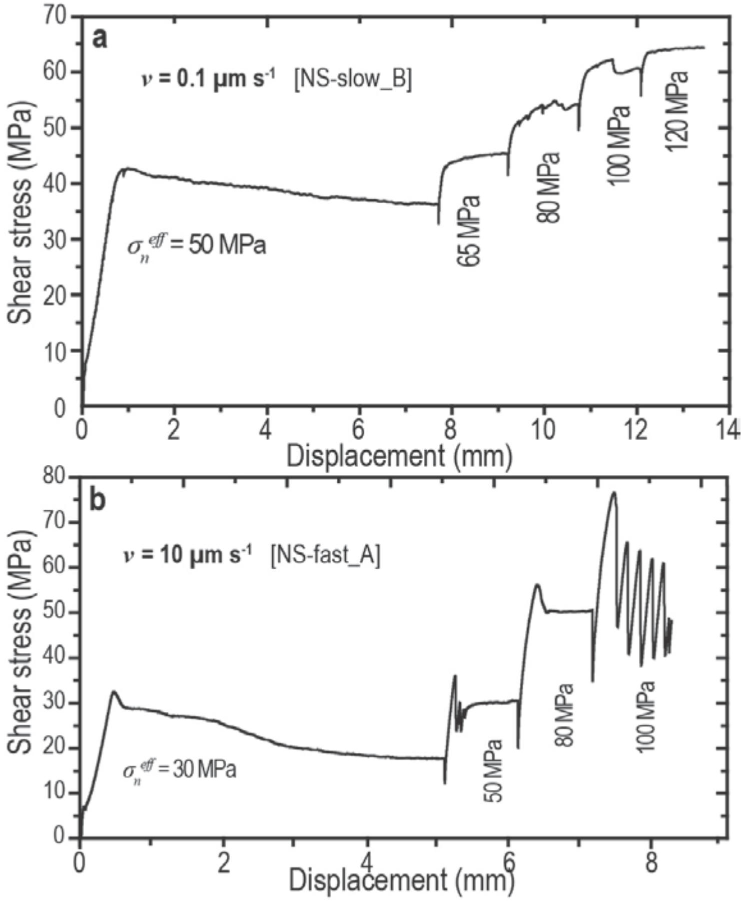

Supplementary Figure 1. Shear stress vs displacement curves effective normal stress stepping experiments. (a) NS-slow_B (0.1 μm $\mathbf{s^{-1}}$) (**b**) NS-fast_A (10 μm $\mathbf{s^{-1}}$). for a list of experiments and strength data see Supplementary Tables 1 and 2.

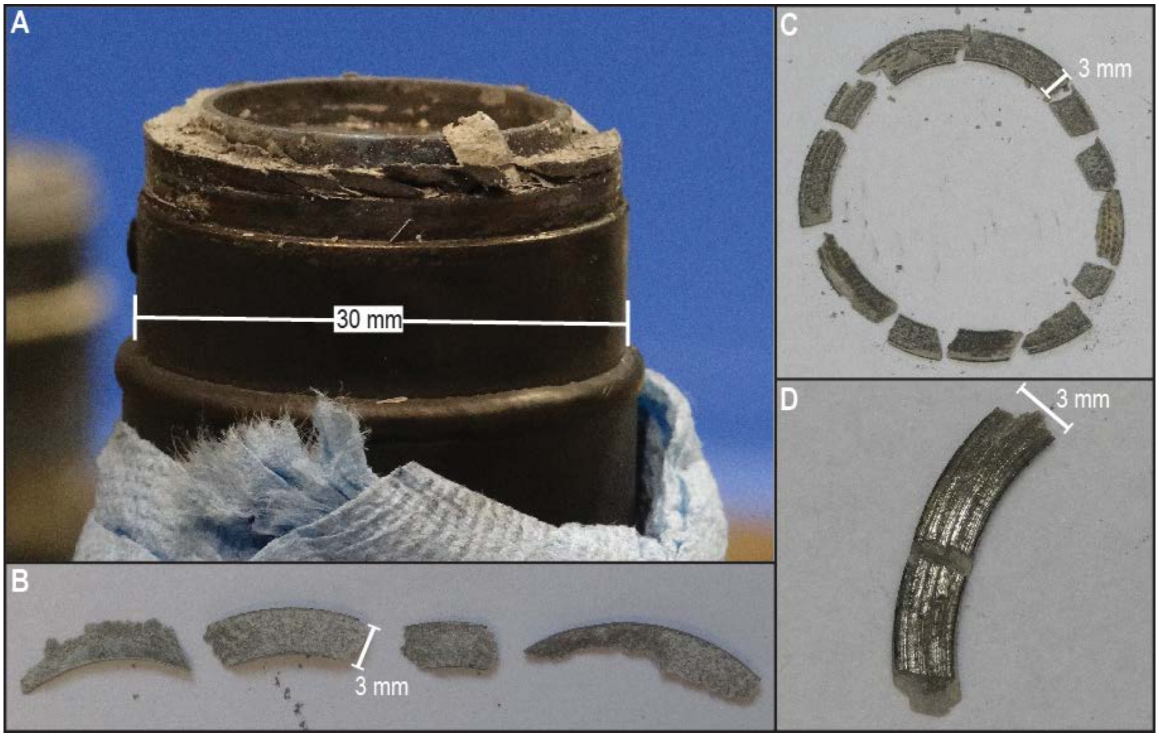

Supplementary Figure 2. Visual inspection of samples after an experiment. (**a**) Lower internal ring shear piston after removal of the outer confining ring. Notice the fractures at a R_1 (Riedel) angle to the shear plane. (**b**) to (**d**) Arc-shaped gouge fragments recovered from experiments at a constant effective normal stress of 50 MPa using a displacement rate (*v*) of 0.1 μm/s ((B), 550-slow) and of 100 μm/s ((c), 550-fast), plus from an effective normal stress stepping experiment performed using v = 10 μm $\mathbf{s^{-1}}$.

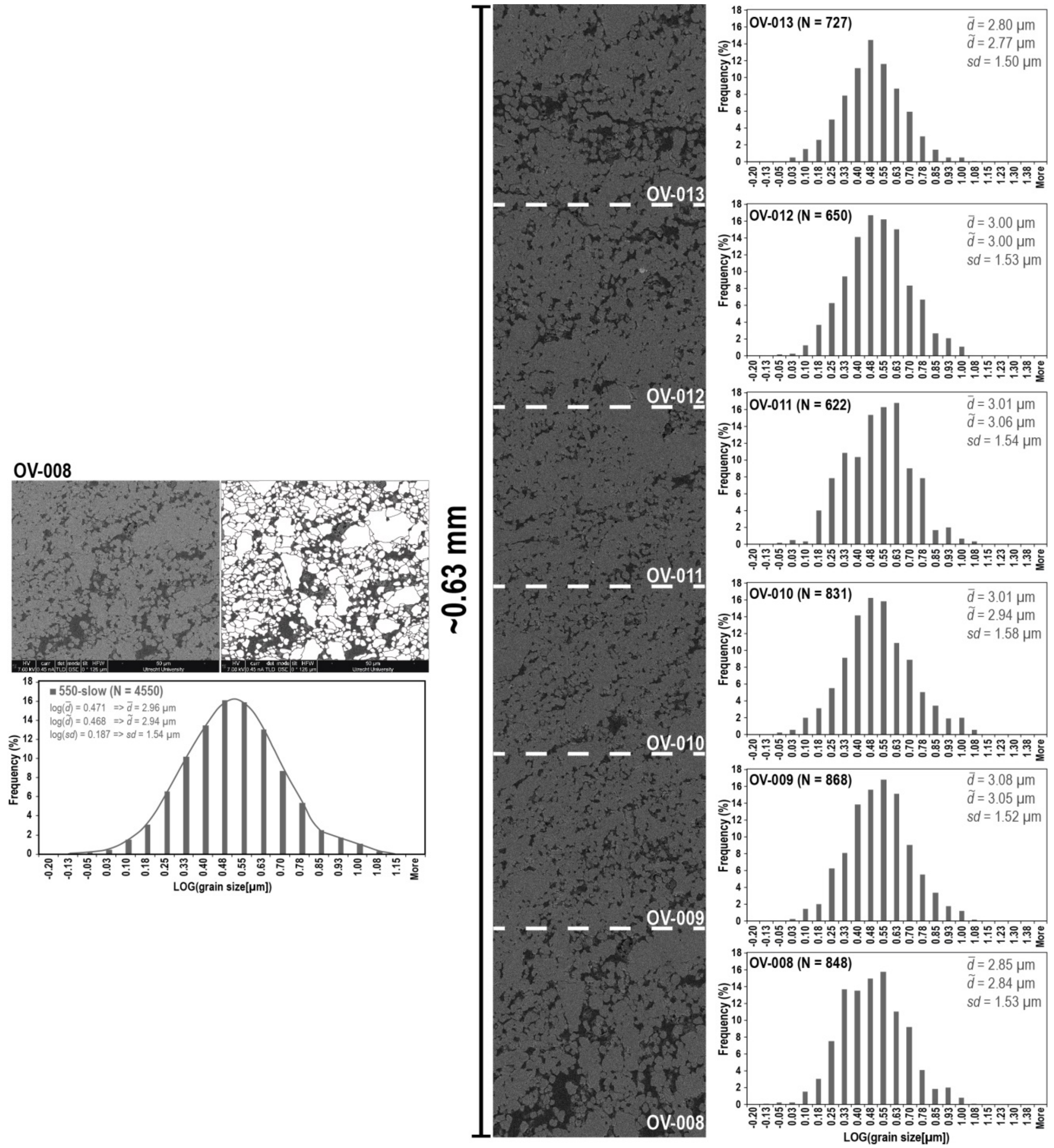

Supplementary Figure 3. Grain size analysis on microstructures recovered from shear test at v = 0.1 μm s^{-1}. (**a**) Grain map, interpreted using ESRI ArcMaps. The histogram shows the grain size distribution (GSD) of all mapped grains (N=4550). (**b**) Vertical transect through sample 550-slow (Fig. 2a), with histograms showing the corresponding GSD’s.

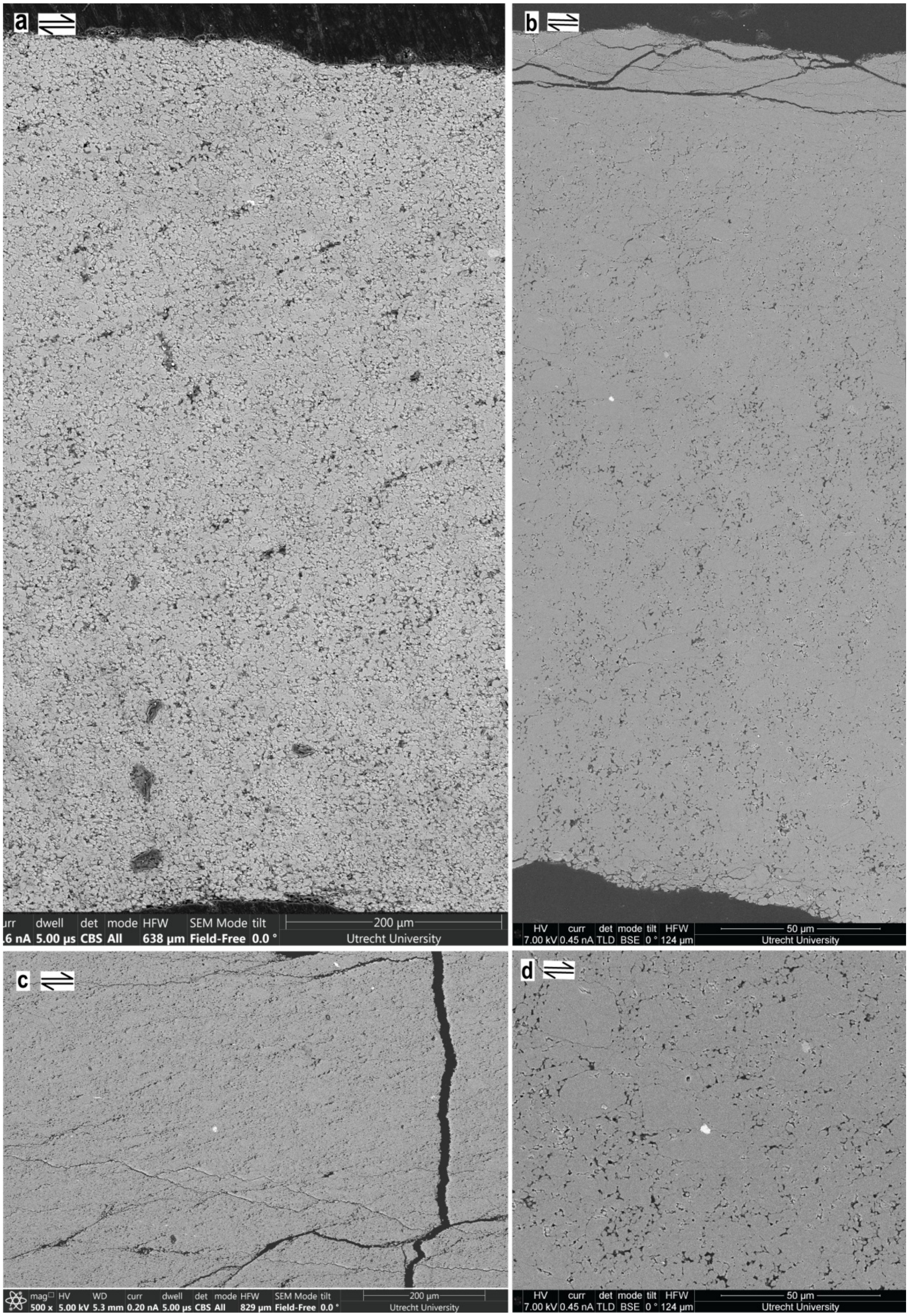

Supplementary Figure 4. Bulk gouge or matrix recovered from shear tests using $v \leq 0.1$ µm s^{-1} and $v = 100$ µm s^{-1}. Microstructures recovered from experiments 550-slow **(a)** VS-slow **(b)**, and 550-fast (**c** and **d**). See Supplementary Table 1 for a list of experiments and conditions.

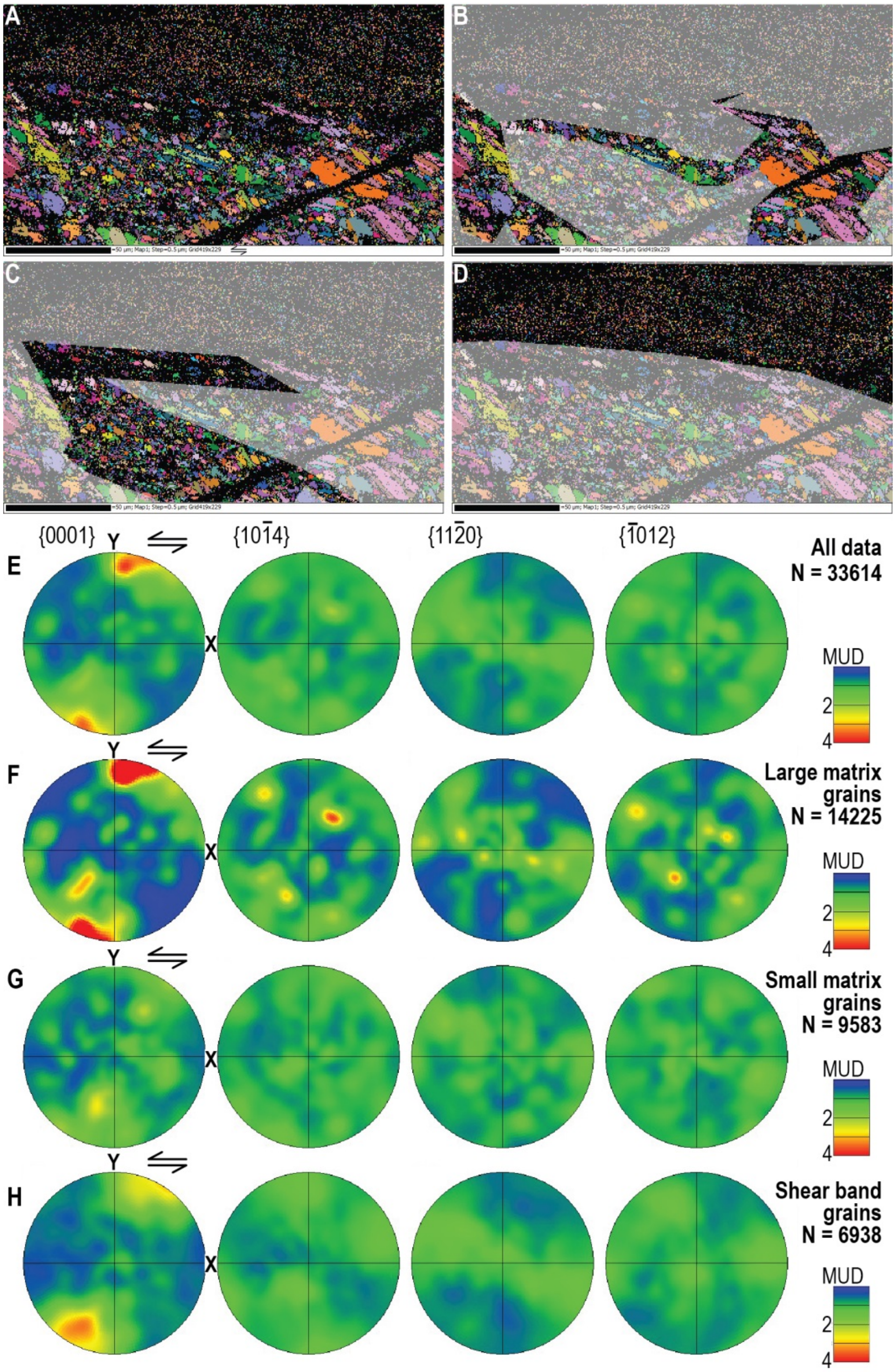

Supplementary Figure 5. Electron backscatter diffraction data of shear band and adjacent grains. Step size 0.5 μm. (**a**) to (**d**) EBSD maps, with Euler angle coloring. Map (a) shows all data. Maps (b) and (c) respectively highlight large and small grains adjacent to the shear band, and map (d) highlights shear band grains. (**e**), (**f**), (**g**), and (**h**) show upper hemisphere, equal angle pole figures of EBSD data corresponding to the highlighted areas in, respectively, (a) to (d). N = the number of data points, MUD = mean uniform density. Pole figures are constructed using a half width of 15º and cluster size of 5º.

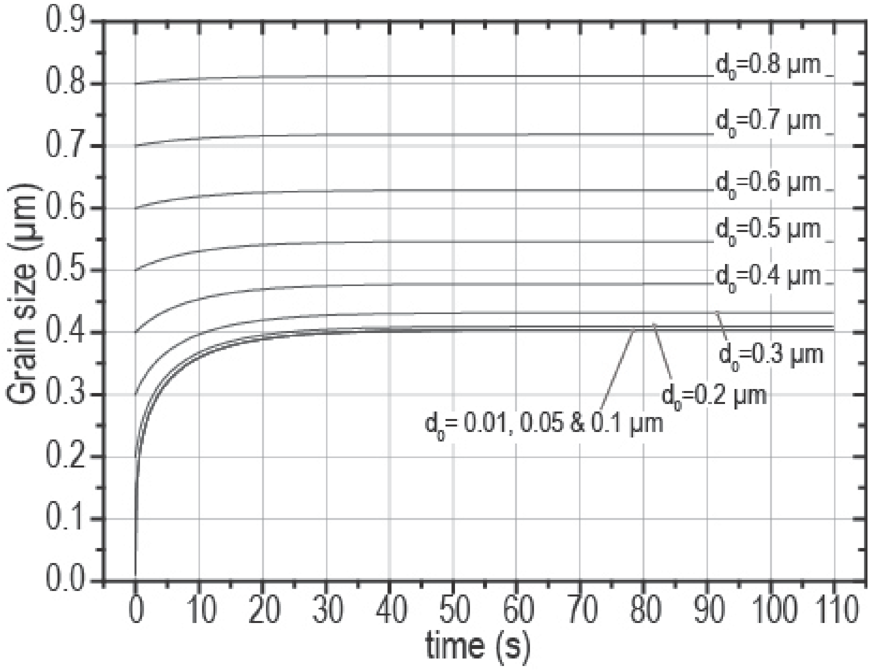

Supplementary Figure 6. Static grain growth upon cooling after our experiments. Based on a generalized grain growth model[49,50], using the kinetics parameters for grain growth in porous (up to 40%), polycrystalline calcite aggregates (grain size ~3 to 5 μm) at 550ºC to 700ºC, under hydrostatic conditions[49]. The plot shows that the predicted evolution of grain size with time for different initial grain sizes d_0.
